# Carbon Nanofibers Propped Hierarchical Porous SiOC Ceramics Toward Efficient Microwave Absorption

**DOI:** 10.1186/s11671-020-3253-y

**Published:** 2020-01-30

**Authors:** Yani Liu, Sifan Zeng, Zhen Teng, Wanlin Feng, Haibin Zhang, Shuming Peng

**Affiliations:** Innovation Research Team for Advanced Ceramics, Institute of Nuclear Physics and Chemistry, China Academy of Engineering Physics, Mianyang, 621900 Sichuan China

**Keywords:** SiOC, Carbon nanofibers, Porous ceramics, Microwave absorption

## Abstract

The hierarchical porous SiOC ceramics (HPSCs) have been prepared by the pyrolysis of precursors (the mixture of dimethicone and KH-570) and polyacrylonitrile nanofibers (porous template). The HPSCs possess hierarchical porous structure with a BET surface area of 51.4 m^2^/g and have a good anti-oxidation property (only 5.1 wt.% weight loss). Owing to the porous structure, the HPSCs deliver an optimal reflection loss value of − 47.9 dB at 12.24 GHz and an effective absorption bandwidth of 4.56 GHz with a thickness of 2.3 mm. The amorphous SiOC, SiO_x_, and free carbon components within SiOC make contributions to enhancing dipolar polarization. Besides, the abundant interfaces between SiOC and carbon nanofibers (CNFs) are favorable for improving interfacial polarization. The conductive loss arisen from cross-linked CNFs can also boost the microwave absorption performance.

## Introduction

With the rapid development of wireless communication technology, the superfluous electromagnetic wave (EMW) has been regarded as new-type pollution, which is harmful for precise instruments, national security, and even human health [1–3]. It is urgent to develop high-performance microwave absorption materials (MAMs) to suppress the undesirable electromagnetic pollution. Recently, porous structures have been proven to be favorable for prolonging propagation paths and then improving microwave scattering, thus leading to a better microwave absorption performance. For instances, Yin et al. presented that the ultra-broad effective microwave band of cellular foam reached 29.7 GHz arising from the well-interconnected porous structure [4]. Li et al. reported that porous carbon delivered a minimum reflection loss (RL_min_) value of − 56.4 dB, which was owing to the improvement of polarization abilities and multiple reflections [5]. Additionally, the porous materials can usually meet the requirement of lightweight for advanced MAMs. Thus, designing a porous structure is an efficient strategy to enhance the MA properties of MAMs.

Among these porous materials, the porous ceramics as rising stars have drawn extensive attention owing to their anti-oxidation, low thermal expansion, and chemical and physical durability characteristics [6, 7]. Therefore, they are strongly relevant for a series of applications, such as catalytic reactor, filtration, thermal energy storage, water treatment, and MAMs [8–11]. According to the previous studies, the SiOC ceramics are considered as promising candidates for MA applications because of their amorphous phases (the complex components of SiOC, SiO_x_, and free carbon), low cost, and lightweight features [12–15]. Benefiting from the existence of free carbon component, the electrical conductivity of SiOC material is much higher than that of SiC (a wide band gap semiconductor), resulting in a higher electronic dipole polarization loss. For example, Yin et al. reported that the RL_min_ value of SiOC ceramics could reach − 46 dB, and the good MA ability was mainly attributed to dipolar polarization occurring in SiC and free carbon phases [14]. However, there are little reports about designing porous SiOC structures for MA applications. Above all, it is expected to develop a facile method to prepare the porous SiOC ceramics as high-performance microwave absorbers.

Herein, the hierarchical porous SiOC ceramics (HPSCs) have been constructed through integrating a simple precursor and non-woven fiber fabric template. The XPS results reveal that the SiOC ceramics are composed of SiOC, SiO_x_, and free carbon. Based on the transmission line theory, the HPSCs deliver an optimal RL value of − 47.9 dB and an effective absorption bandwidth (EAB) of 4.56 GHz. The good MA performance is attributed to multiple reflections, diversified polarization, and conductive losses. This facile approach can open a new avenue toward the fabrication of polymer-derived porous ceramics for MA applications.

## Experimental Methods

### Synthesis of HPSCs

For HPSC preparation, the dimethicone (Sinopharm Chemical Reagent) and KH-570 (Sinopharm Chemical Reagent) were used as raw materials to prepare the precursor. Firstly, they were mixed with a weight ratio of 19:1 and then stirred at 80 °C for 6 h. Secondly, the non-woven fiber fabrics were used as templates via an electrospinning method. One gram of polyacrylonitrile (PAN; Macklin) powder was dissolved in 9.0 g *N*,*N*-dimethylformamide (DMF; Sinopharm Chemical Reagent) solvent with stirring for 5 h. Subsequently, the electrospinning was performed at a voltage of 18 kV and a feeding rate of 10 μL/min. To obtain the precursor/PAN hybrid, the as-prepared precursor was injected into PAN fabrics. Finally, the hybrid was heated to 1000 °C for 2 h at a heating rate of 2 °C/min under argon atmosphere. After cool down, the HPSCs were collected without any further treatment.

### Characterization

The morphologies of the samples were investigated by field-emission scanning electron microscopy (FESEM; FEI Apreo). The X-ray photoelectron spectroscopy (XPS, Thermo-VG Scientific, ESCALAB 250) was used with a monochromatic Al-Kα X-ray source (excitation energy = 1486 eV). The Raman spectra were tested through a microscopic confocal Raman spectrometer (Renishaw RM2000) with a wavelength of 514 nm at room temperature. The compositions of the sample were studied by X-ray diffraction (XRD) by a Rigaku D/max-RB12 X-ray diffractometer with Cu Kα radiation. The thermogravimetry analysis (TGA) was recorded on a TGA/Q5000IR analyzer under ambient atmosphere. The nitrogen adsorption and desorption isotherms were measured by ASAP 2020 Accelerated Surface Area and Porosimetry instrument.

### Microwave Absorption Measurement

The electromagnetic parameters of samples mixed with wax (50 wt.%) were measured at 2~18 GHz using Vector network analyzer (N5245A, Agilent). The reflection loss (RL) values were calculated based on transmission line theory using the following equations [16, 17].

1 $$ {Z}_{\mathrm{in}}={Z}_0{\left({\mu}_r/{\varepsilon}_r\right)}^{1/2}\tanh \left[j\left(2\pi fd/c\right){\left({\mu}_r/{\varepsilon}_r\right)}^{1/2}\right] $$

2 $$ RL=20\log \mid \left({Z}_{\mathrm{in}}-{Z}_0\right)/\left({Z}_{\mathrm{in}}+{Z}_0\right)\mid $$

where *ε*_*r*_ and *μ*_*r*_ are the relative complex permittivity and permeability respectively, *f* is the frequency of microwave, *d* is the thickness of samples, *c* is the velocity of microwave in free space, *Z*_in_ is the lumped input impedance at the absorber surface, and *Z*_0_ is the characteristic impedance of air [18].

## Results and Discussion

**Fig. 1:**
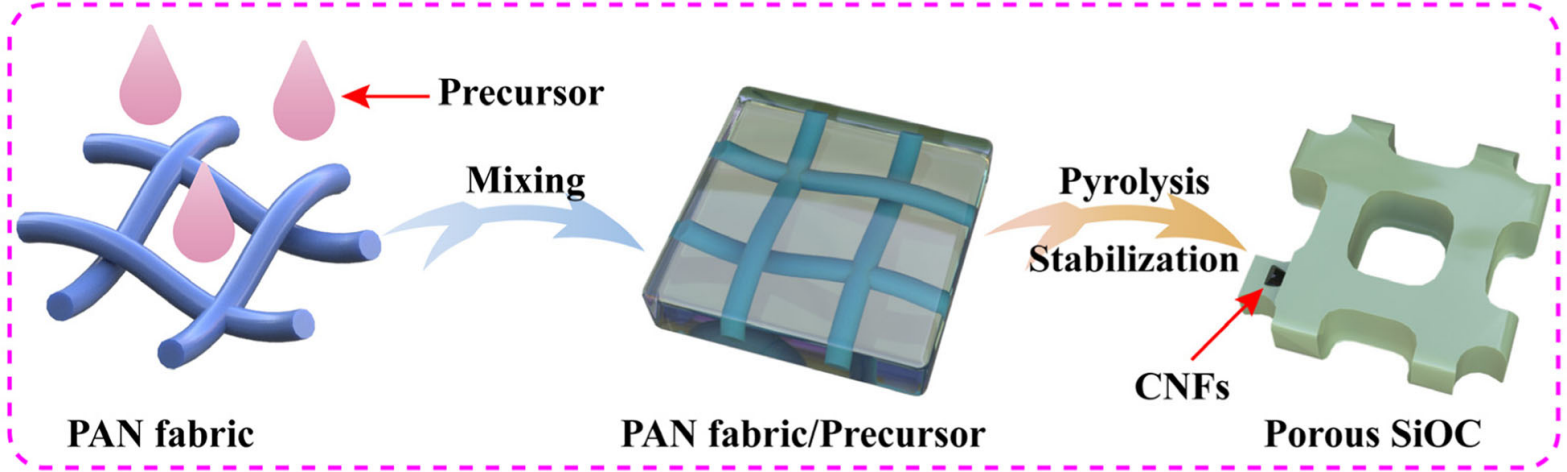
The schematic illustration of fabrication of HPSCs

**Fig. 2:**
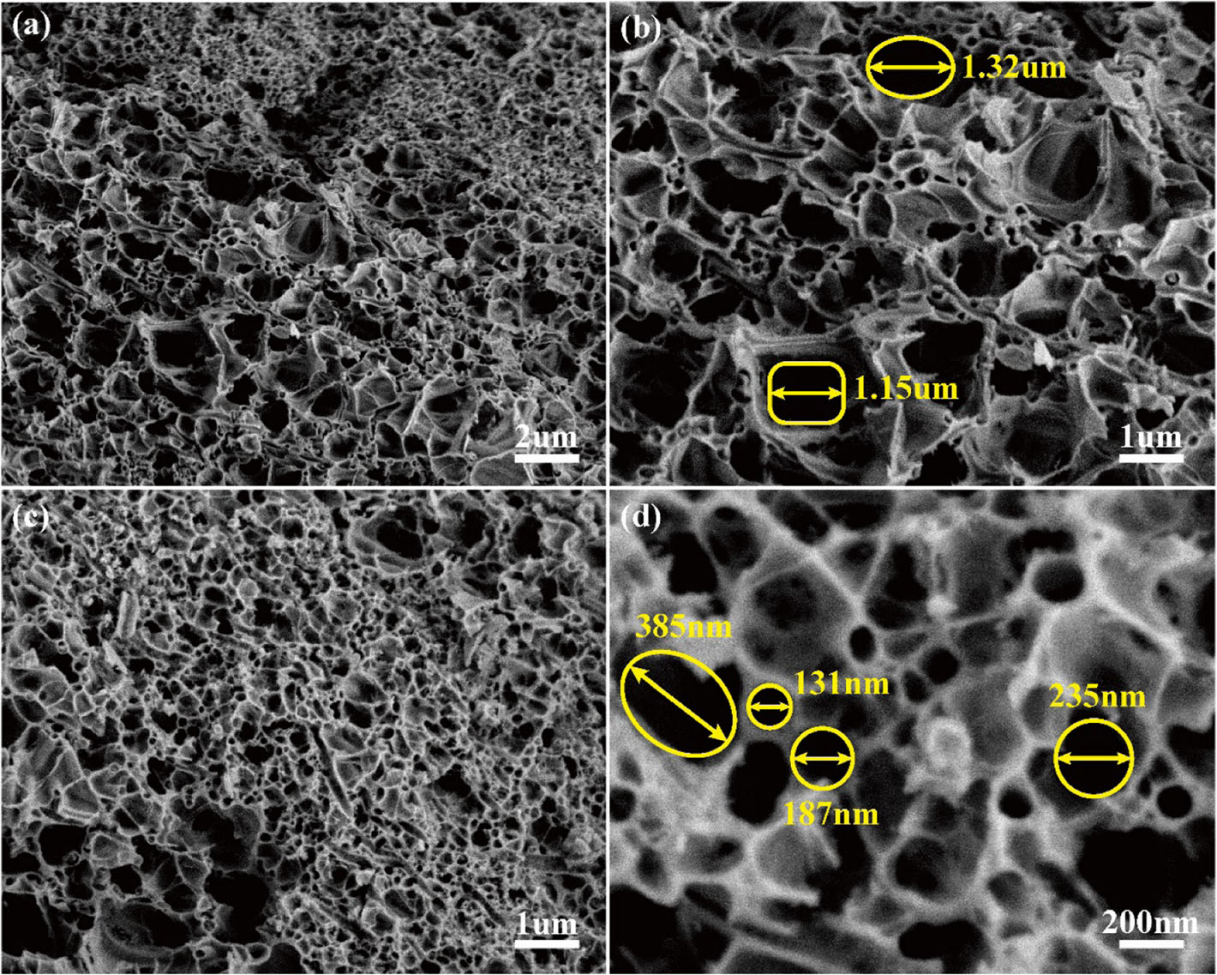
The SEM images of HPSCs at different magnification: **a** × 5.0 k, **b** × 10.0 k, **c** × 10.0 k, and **d** × 50.0 k

Figure 1 shows the schematic illustration of fabrication of HPSCs. Step 1: the precursor was prepared by dimethicone and KH-570, and the PAN nanofiber fabric was obtained via an electrospinning method. Additional file 1: Figure S1 shows the optical image of PAN fabric (8 cm × 14 cm). Additional file 1: Figure S2 exhibits the cross-linked PAN nanofibers with a diameter of 378 nm. These cross-linked fibers form a large number of pores, which can be directly used as porous templates. Step 2: the as-prepared precursor was injected into PAN fabrics. Step 3: the HPSCs were obtained after a heat treatment. After pyrolysis and stabilization, the precursor and PAN nanofibers were transformed to SiOC ceramics and carbon nanofibers (CNFs), respectively. The CNFs were regarded as the backbone to prop porous structure, and the SiOC ceramics wrapped onto the surface of CNFs. Thus, the HPSCs were formed through a template/precursor pyrolysis method. As shown in Fig. 2a, the HPSCs exhibit a large number of pores with hierarchical porous structures. Figure 2b displays the irregular pores with a size of 1.2 μm, corresponding to the escape of gas (CH_4_, H_2_) in the precursor pyrolysis process. Figure 2 c and d exhibit much more uniform pores with a size of 200 nm, which are mainly constructed by the cross-linked carbon nanofibers.

**Fig. 3:**
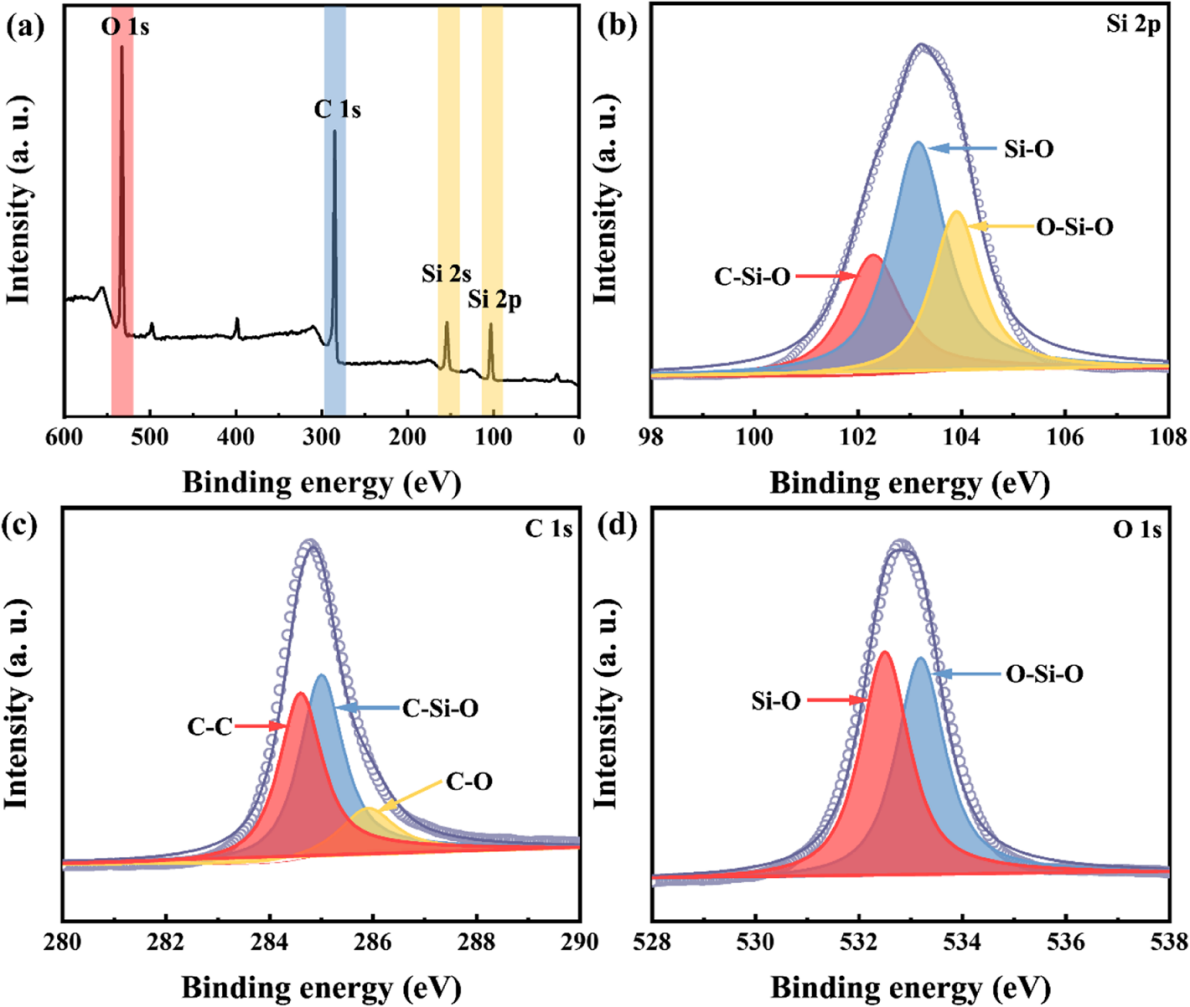
The XPS spectra of HPSCs. **a** The survey spectrum. **b** The fitted Si 2p peak. **c** The fitted C 1s peak. **d** The fitted O 1s peak

The XPS spectra (Fig. 3) are performed to verify the composition of HPSC samples. The survey spectrum (Fig. 3a) ascertains the existence of Si, C, and O elements within the HPSC sample. As shown in Fig. 3b, the broad peak of Si 2p exhibits three fitted bands around at 102.30, 103.15, and 103.90 eV, corresponding to C–Si–O, Si–O, and O–Si–O bonds, respectively [19]. The higher binding energy of 103.90 eV for O–Si–O bond is mainly attributed to the higher electronegativity of O atom (3.610) than those of C (2.544) and Si (1.916) atoms. As shown in Fig. 3c, the spectrum of C 1s displays the presence of different valence around C atom originating from bonding with other elements. It can be divided into three bands at 284.60, 285.00, and 285.90 eV, which are related to C–C, C–Si–O, and C–O bonds, respectively [20]. Figure 3d reveals that the fitted O 1s band suggests the presence of Si–O (532.50 eV) and O–Si–O (533.20 eV) bonds. The XPS results indicate that the SiOC component has been successfully obtained via this precursor pyrolysis method.

**Fig. 4:**
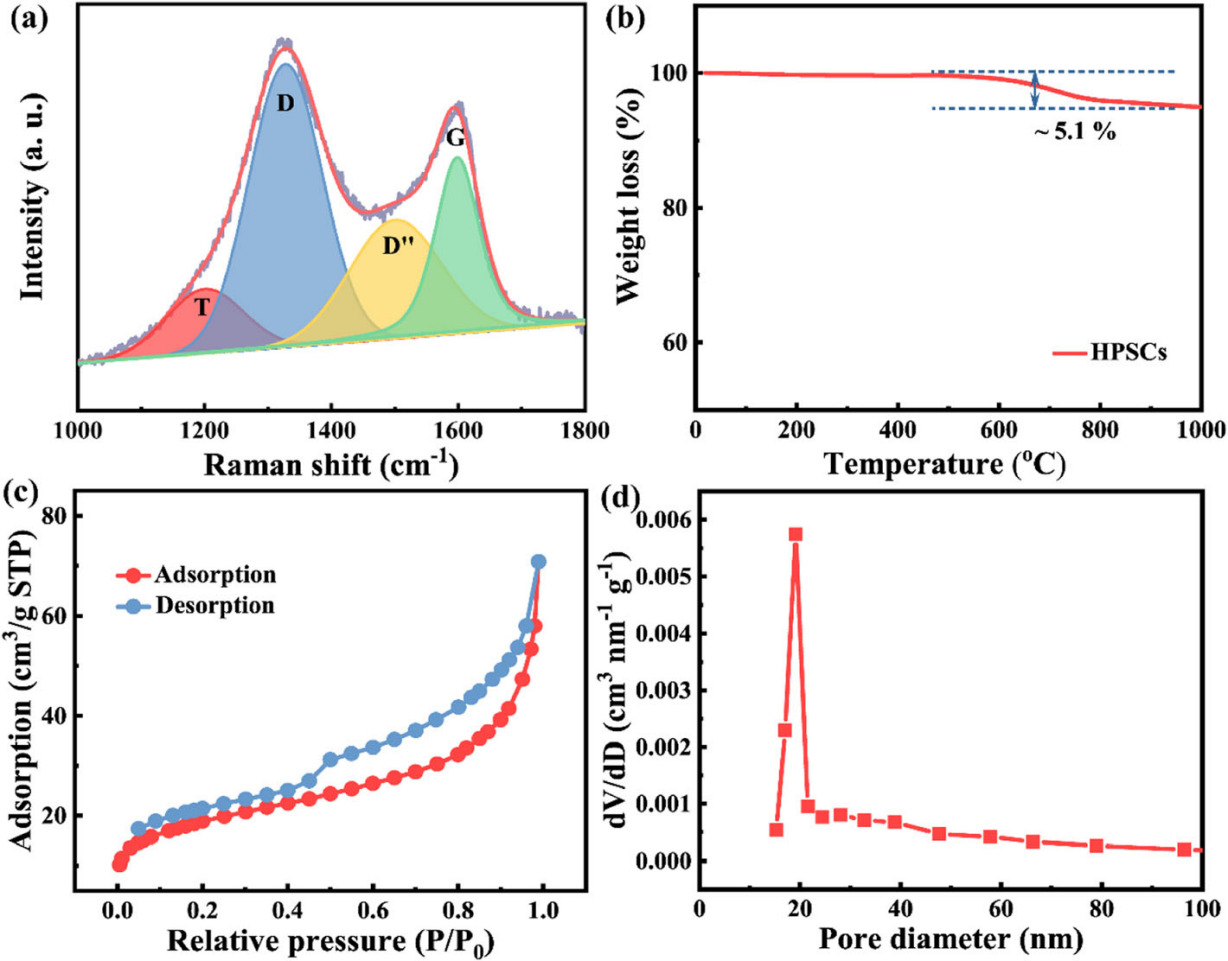
**a** The Raman spectrum. **b** TGA curve under air atmosphere. **c** N_2_ adsorption–desorption curves. **d** Pore size distribution of HPSC samples

The Raman spectrum (Figure 4a) was carried out to ascertain the existence of free carbon phase within SiOC ceramics. The Raman spectrum can be fitted into D, G, T, and D” bands. The typical D and G bands are located at 1328 and 1598 cm^−1^, indicating the amorphous carbon structure. The D and T bands are ascribed to electron–hole relaxation originating from disordered graphitic carbon, while the D” band is associated with amorphous carbon soot. And the G band is corresponding to E_2g_ mode arising from in-plane stretching vibration of sp^2^ hybridized bonds [21]. The XRD pattern of HPSCs is plotted in Additional file 1: Figure S3. A broad peak around at 24.5° is mainly attributed to the amorphous carbon phase within SiOC ceramics and PAN-derived carbon nanofibers [22, 23]. The TGA characterization was carried out to measure the anti-oxidation property of HPSCs. Figure 4b shows the TGA curve in the temperature of 20~1000 °C under flowing air atmosphere. A weak weight loss is about 5.1 wt.% in the range of 450~800 °C, which is attributed to the oxidation of free carbon component within SiOC ceramics. Based on the TGA result, it can be concluded that HPSCs show good thermal stability and anti-oxidation properties, and carbon fibers as template have been totally wrapped and protected by SiOC ceramics. The N_2_ adsorption–desorption isotherms are performed to investigate Brunauer–Emmet–Teller (BET) surface area of HPSCs. Figure 4c shows a typical type IV behavior, revealing the presence of mesopores in HPSC samples. And the HPSCs deliver a BET surface area of 51.4 m^2^/g. The pore size distribution is studied by the Barrett–Joyner–Halenda (BJH) model. Figure 4d shows that HPSCs also possess a lot of mesopores with a diameter of 20 nm.

**Fig. 5:**
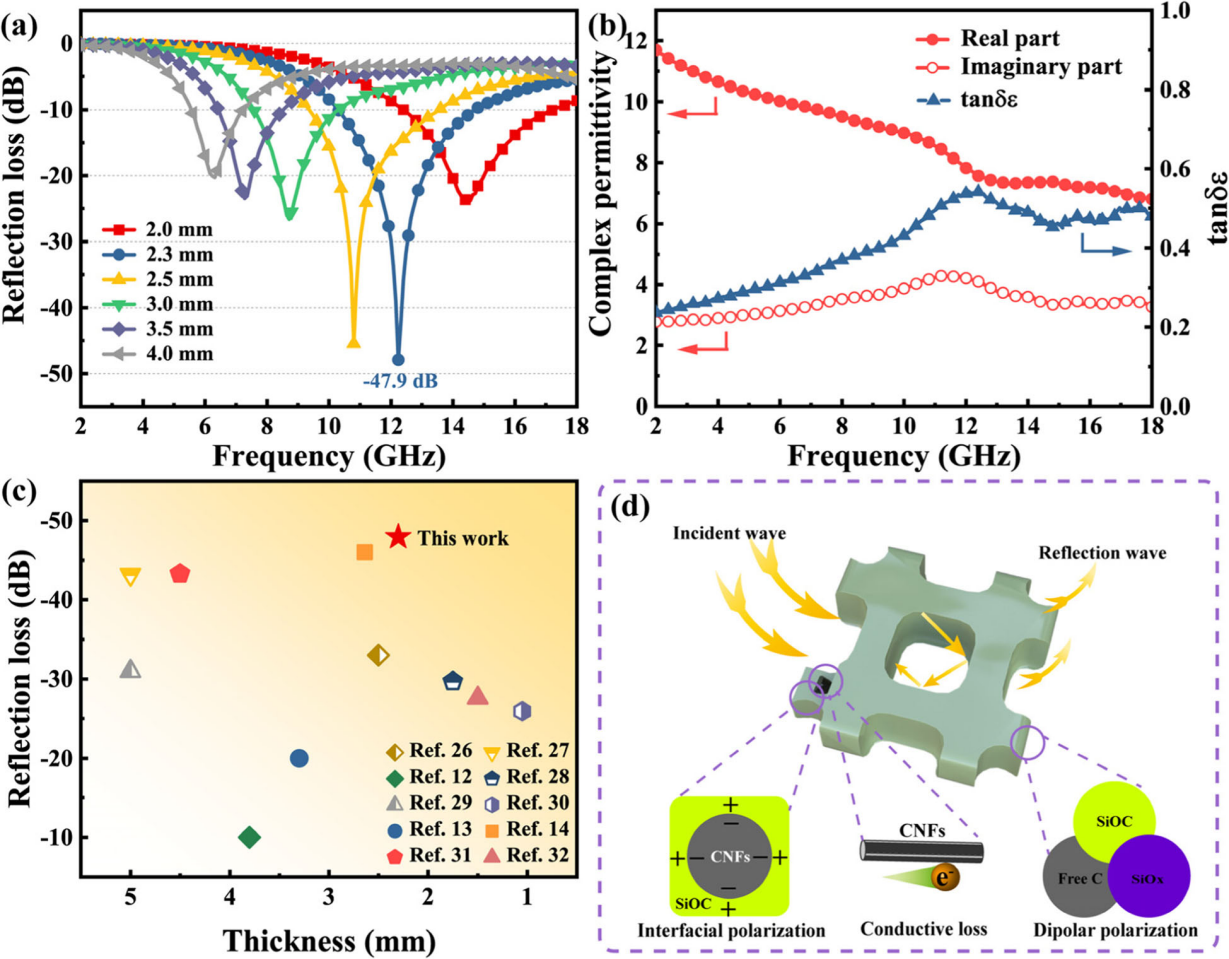
The MA properties of HPSCs. **a** The RL curves. **b** The complex permittivity and tangent loss curves. **c** RL_min_ versus thickness of similar Si-based ceramics absorbers. **d** The schematic illustration of MA mechanism

As shown in Fig. 5a, the MA performance of HPSCs is illustrated by the RL curves versus frequency at different layer thickness. The HPSCs deliver an optimal RL_min_ value of − 47.9 dB at 12.24 GHz, and an EAB of 4.56 GHz in the range of 10.24~14.8 GHz with a matching thickness of 2.3 mm. The RL_min_ values can reach − 23.8 dB at 14.56 GHz, − 47.9 at 12.24 GHz, − 45.5 at 10.8 GHz, − 26.6 at 8.72 GHz, − 23.5 at 7.28 GHz, and − 20.3 dB at 6.32 GHz with the thicknesses of 2.0, 2.3, 2.5, 3.0, 3.5, and 4.0 mm, respectively. This phenomenon can be interpreted by the quarter-wavelength cancelation model, which illustrates the relationship between matching thickness (*t*_m_) and corresponding matching frequency (*f*_m_) by the following equation [24, 25].

3 $$ {t}_{\mathrm{m}}= n\lambda /4= n c/\left(4\ {f}_{\mathrm{m}}\ \sqrt{\left|{\varepsilon}_r\right|\left|{\mu}_r\right|}\right)\kern1.25em n=\left(1,3,5,\dots \right) $$

When the *t*_m_ and *f*_m_ meet Eq. (3) well, the phase difference between the incident wave and the reflective wave is 180°, which means that the RL_min_ can be obtained owing to the dissipation of electromagnetic energy at the air-absorber interface [26]. Additional file 1: Figure S4 shows the *t*_m_ versus *f*_m_ curves of 1*λ*/4 for HPSCs; it is apparent that ${t}_{\mathrm{m}}^{\mathrm{exp}}$ dots are located at the ${t}_{\mathrm{m}}^{\mathrm{cal}}$ lines, revealing that this model can expound the relationship between *t*_m_ and *f*_m_ well. The complex permittivity is tightly relevant to MA performance, and the tangent loss (tan*δε* = *ε*″/*ε*′) is generally used to evaluate the attenuating ability of MAMs [27]. The real part (*ε*′) represents the storage ability of EM energy, while the imaginary part (*ε*″) is corresponding to the loss ability of EM energy [28]. Figure 5b displays the complex permittivity and tan*δε* curves of HPSCs. The *ε*′ declines in the whole range, and the *ε*″ delivers a peak in the range of 9.2~13.6 GHz. Therefore, the tan*δε* exhibits a relaxation peak around at 12.0 GHz, which is close to that (12.24 GHz) of the optimal RL_min_. As shown in Additional file 1: Figure S5, the real and imaginary parts of complex permeability are nearly equal to 1 and 0, respectively, which is ascribed to the non-magnetism of HPSCs. Figure 5c shows a comparison of RL_min_ value versus thickness of similar Si-based ceramics materials in recent studies [12–14, 29–35]. Additional file 1: Table S1 lists the detailed MA data of all related references. It can be found that the HPSCs not only deliver an optimal RL value but also possess a thin thickness.

4 $$ \alpha =\frac{\sqrt{2}\pi f}{c}\times \sqrt{\left({\mu}^{\prime \prime }{\varepsilon}^{\prime \prime }-\mu^{\prime}\varepsilon^{\prime}\right)+\sqrt{\left({\mu}^{\prime \prime }{\varepsilon}^{\prime \prime }-\mu^{\prime}\varepsilon^{\prime}\right)+\left({\mu}^{\prime \prime }{\varepsilon}^{\prime }+\mu^{\prime}\varepsilon^{\prime\prime}\right)}} $$

Generally, the EM attenuation constant (*α*) is regarded as an important factor to assess the dissipation capability, and it can be expressed by Eq. (4) [36]. As shown in Additional file 1: Figure S6, the HPSCs show an increasing trend and strong attenuation ability in the range of 2~18 GHz. These values are much larger than those of similar Si-based materials [31, 33]. On the other hand, a proper impedance matching is favorable to make more microwave propagate into materials. When the value of |*Z*_in_*/Z*_0_| is equal to 1, it means that there is no any reflection of an incident wave at the air-absorber surface [37]. As shown in Additional file 1: Figure S7, the |*Z*_in_*/Z*_0_| values of HPSCs are close to 1 in the most range of 2~18 GHz. And the optimal RL_min_ value of − 47.9 dB is obtained at 12.24 GHz, and the corresponding |*Z*_in_*/Z*_0_| value (0.994) is nearly equal to 1. Figure 5d demonstrates a possible MA mechanism of HPSCs. Firstly, the porous structure can make contributions to extend the scattering of EMW, enhancing the attenuation of electromagnetic energy [5]. Secondly, the dipolar polarization is arisen from SiOC owing to the existence of SiOC, SiO_x_, and free carbon [38]. And there are a large amount of grain boundaries within the amorphous SiOC structure; it is a benefit to enhancing interfacial polarization. Thirdly, the abundant interfaces between CNFs and SiOC play a vital role in boosting the interfacial polarization [39]. Fourthly, the cross-linked CNFs can provide a continuous transport path for free electrons, which is favorable for enhancing the conductive loss [26, 40]. The proper impedance matching of HPSCs reveals that more microwave can propagate into absorbers, and thus, more electromagnetic energy can be dissipated and converted into heat or other energy. Based on these aspects, the HPSCs exhibit an impressive MA performance. And the MA properties can be optimized by tuning the chemical compositions of SiOC and porous structure (pore size, pore volume).

## Conclusion

In summary, the HPSCs have been successfully obtained via a CNF template method. The SEM images and BET results reveal the hierarchical porous structure of SiOC sample. The XPS results indicate that SiOC is formed by SiOC, SiO_x_, and free carbon components. The HPSCs show good anti-oxidation property according to the result of TGA. The optimal RL value and EAB of HPSCs can reach − 47.9 dB and 4.56 GHz at the thickness of 2.3 mm, which is advanced among these similar MAMs. The excellent MA property is originated from multiple reflection, polarization, conductive losses, and favorable impedance matching effect. The HPSCs can be prospective candidates for high-temperature MA application owing to its good anti-oxidation and MA properties.

## Supplementary information

**Additional file 1: Figure S1.** The optical image of PAN fabric. **Figure S2.** The SEM images of electrospinning derived PAN fibers. **Figure S3.** The XRD pattern of HPSCs. **Figure S4.** The reflection loss curves (upper region) and the dependence of matching thickness (*t*_*m*_) on matching frequency (*f*_*m*_) at the wavelength of 1/4λ (lower region) of HPSCs. **Figure S5.** The complex permeability curves of HPSCs. **Figure S6.** The attenuation constant of HPSCs. **Figure S7.** The |*Z*_*in*_*/Z*_*0*_| curve of HPSCs. Table S1. The MA properties of similar Si-based materials

## Data Availability

The data supporting the conclusions of this article are included within the article and its additional files.

## Abbreviations

BET: Brunauer–Emmet–Teller
BJH: Barrett–Joyner–Halenda
CNFs: Carbon nanofibers
DMF: Dimethylformamide
EAB: Effective absorption bandwidth
EMW: Electromagnetic wave
FESEM: Field-emission scanning electron microscopy
HPSCs: Hierarchical porous SiOC ceramics
MAMs: Microwave absorption materials
PAN: Polyacrylonitrile
RL_min_: Minimum reflection loss
TGA: Thermogravimetry analysis
XPS: X-ray photoelectron spectroscopy
