# Supplementary material for: Carbon Nanofibers Propped Hierarchical Porous SiOC Ceramics Toward Efficient Microwave Absorption
Source: Nanoscale Res Lett. 2020 Jan 30;15:28. doi: 10.1186/s11671-020-3253-y (PMC6992838; doi:10.1186/s11671-020-3253-y)
Supplement: Supplementary file 1 — Additional file 1: Figure S1. The optical image of PAN fabric. Figure S2. The SEM images of electrospinning derived PAN fibers. Figure S3. The XRD pattern of HPSCs. Figure S4. The reflection loss curves (upper region) and the dependence of matching thickness (tm) on matching frequency (fm) at the wavelength of 1/4λ (lower region) of HPSCs. Figure S5. The complex permeability curves of HPSCs. Figure S6. The attenuation constant of HPSCs. Figure S7. The |Zin/Z0| curve of HPSCs. Table S1. The MA properties of similar Si-based materials [file 11671_2020_3253_MOESM1_ESM.docx]

Supplementary information

**Carbon Nanofibers Propped Hierarchical Porous SiOC Ceramics toward Efficient Microwave Absorption**

*Yani Liu^1,^*^‡^*, Sifan Zeng^1,^*^‡^, *Zhen Teng^1^, Wanlin Feng^1^, Haibin Zhang^1,^*, Shuming Peng^1,2,^**

^1^Innovation Research Team for Advanced Ceramics, Institute of Nuclear Physics and Chemistry, China Academy of Engineering Physics, Mianyang, Sichuan, 621900, China. E-mail: hbzhang@caep.cn, pengshuming@caep.cn

* Author to whom correspondence should be addressed.

^‡^ Yani Liu and Sifan Zeng contributed equally to this work.


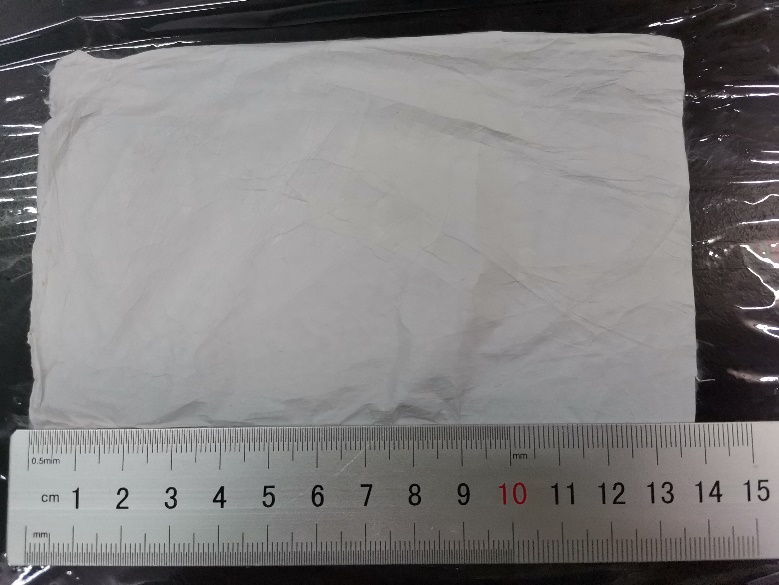


**Figure S1.** The optical image of PAN fabric.


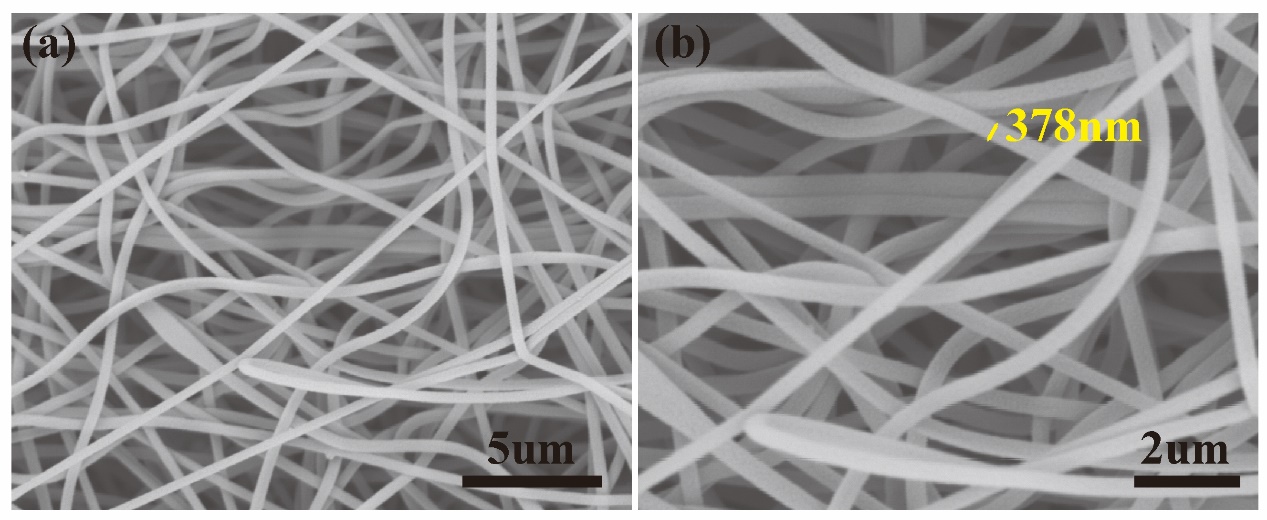


**Figure S2.** The SEM images of electrospinning derived PAN fibers.


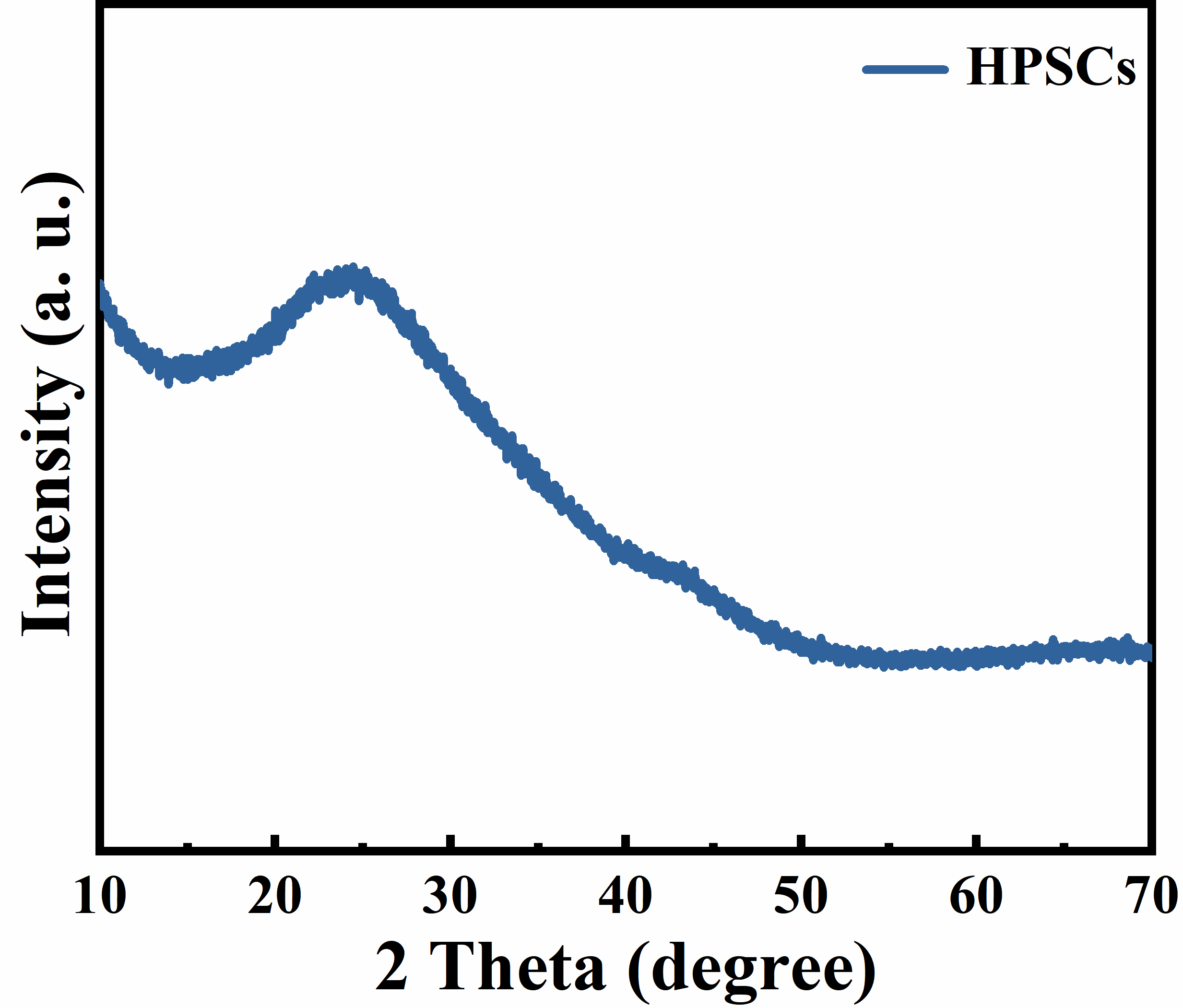


**Figure S3.** The XRD pattern of HPSCs.


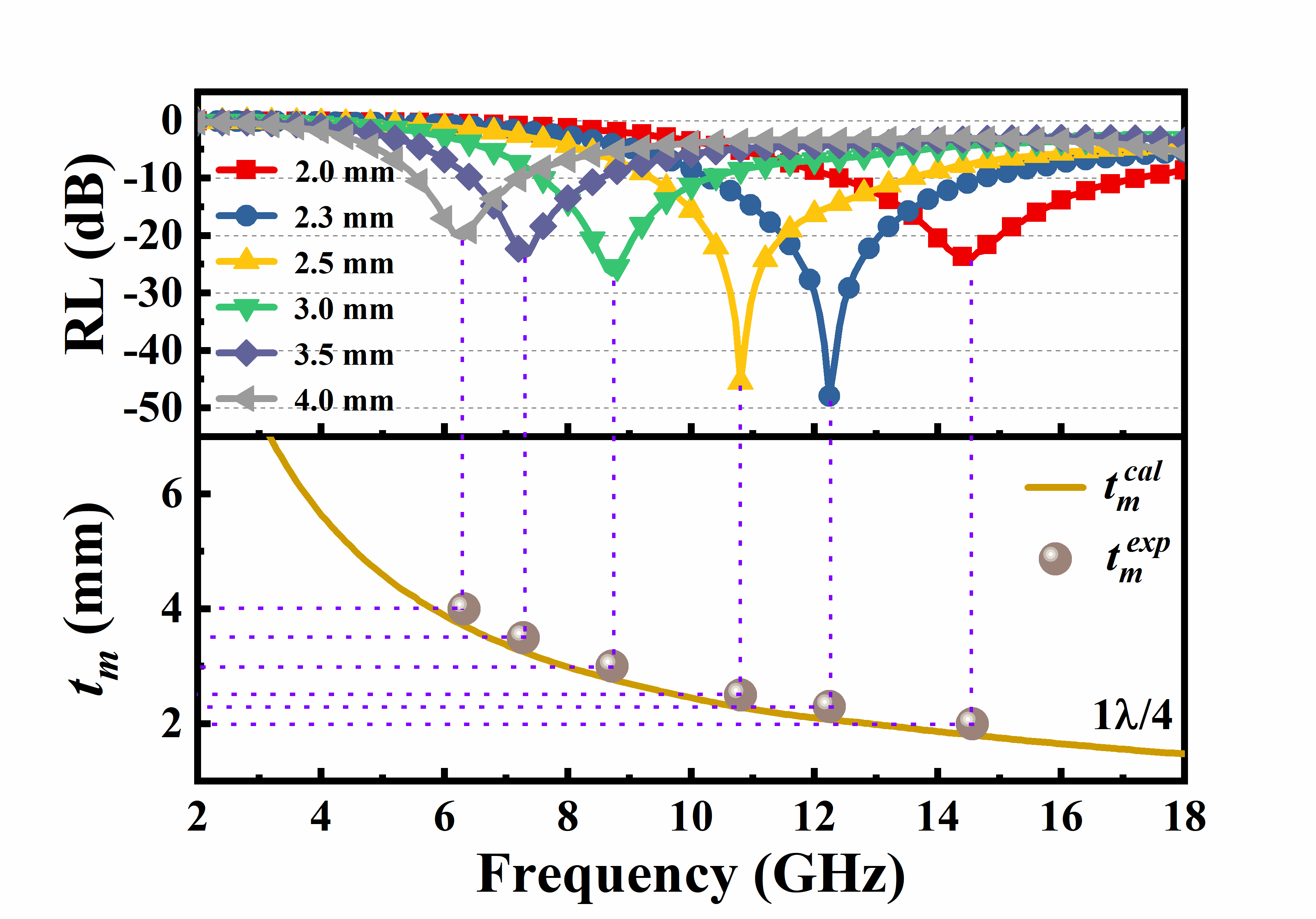


**Figure S4.** The reflection loss curves (upper region) and the dependence of matching thickness (*t_m_*) on matching frequency (*f_m_*) at the wavelength of 1/4λ (lower region) of HPSCs.


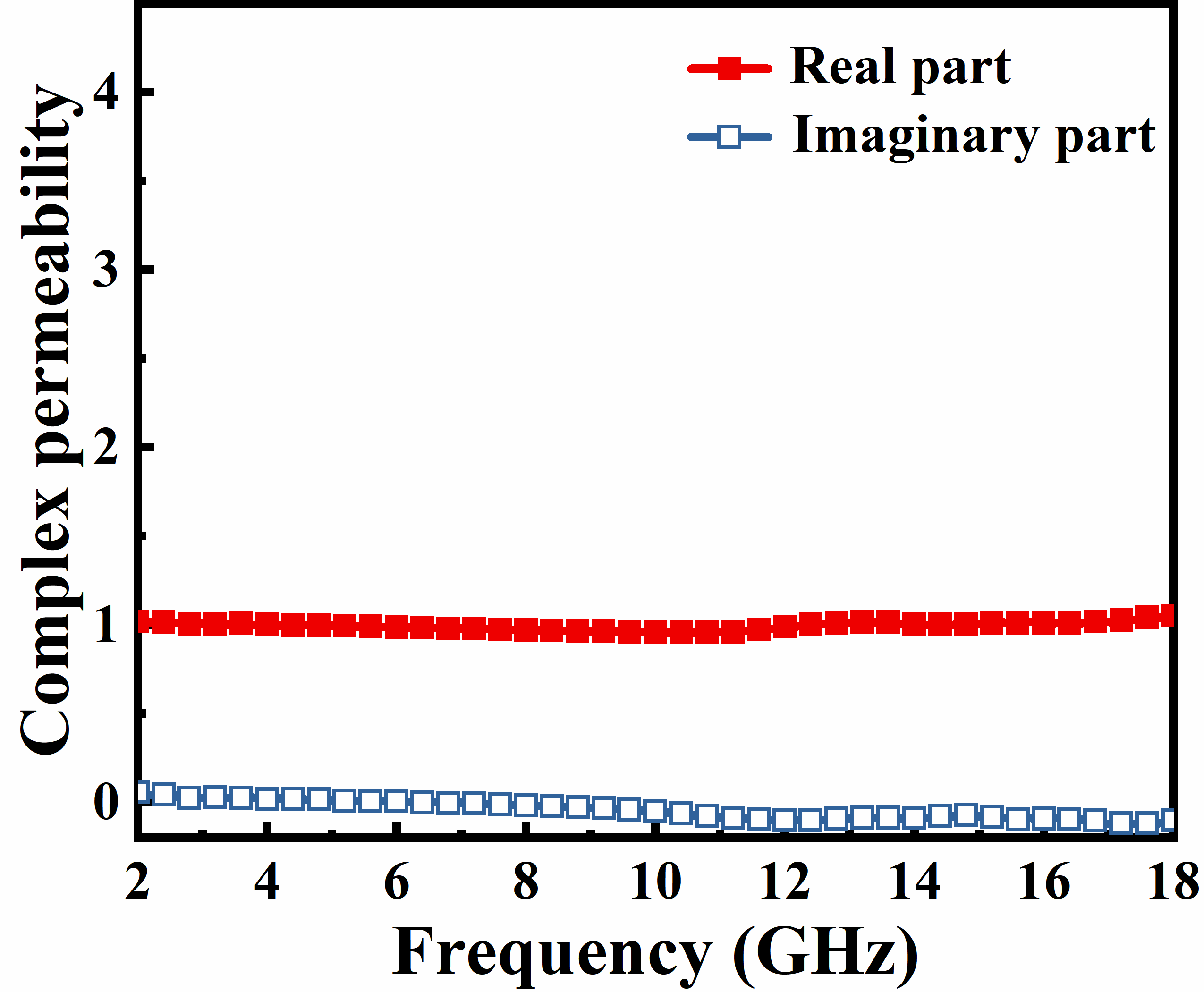


**Figure S5.**The complex permeability curves of HPSCs.


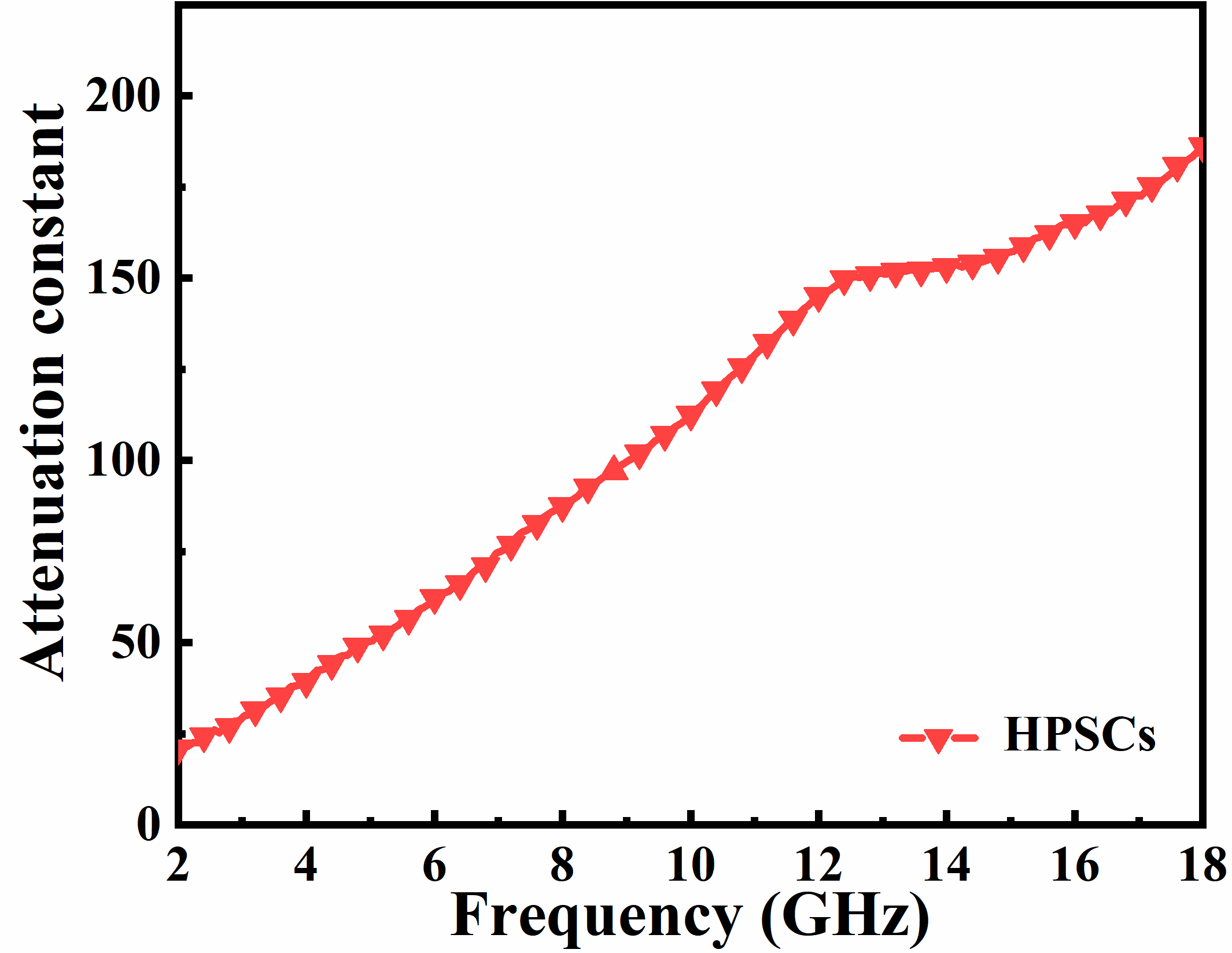


**Figure S6.** The attenuation constant of HPSCs.


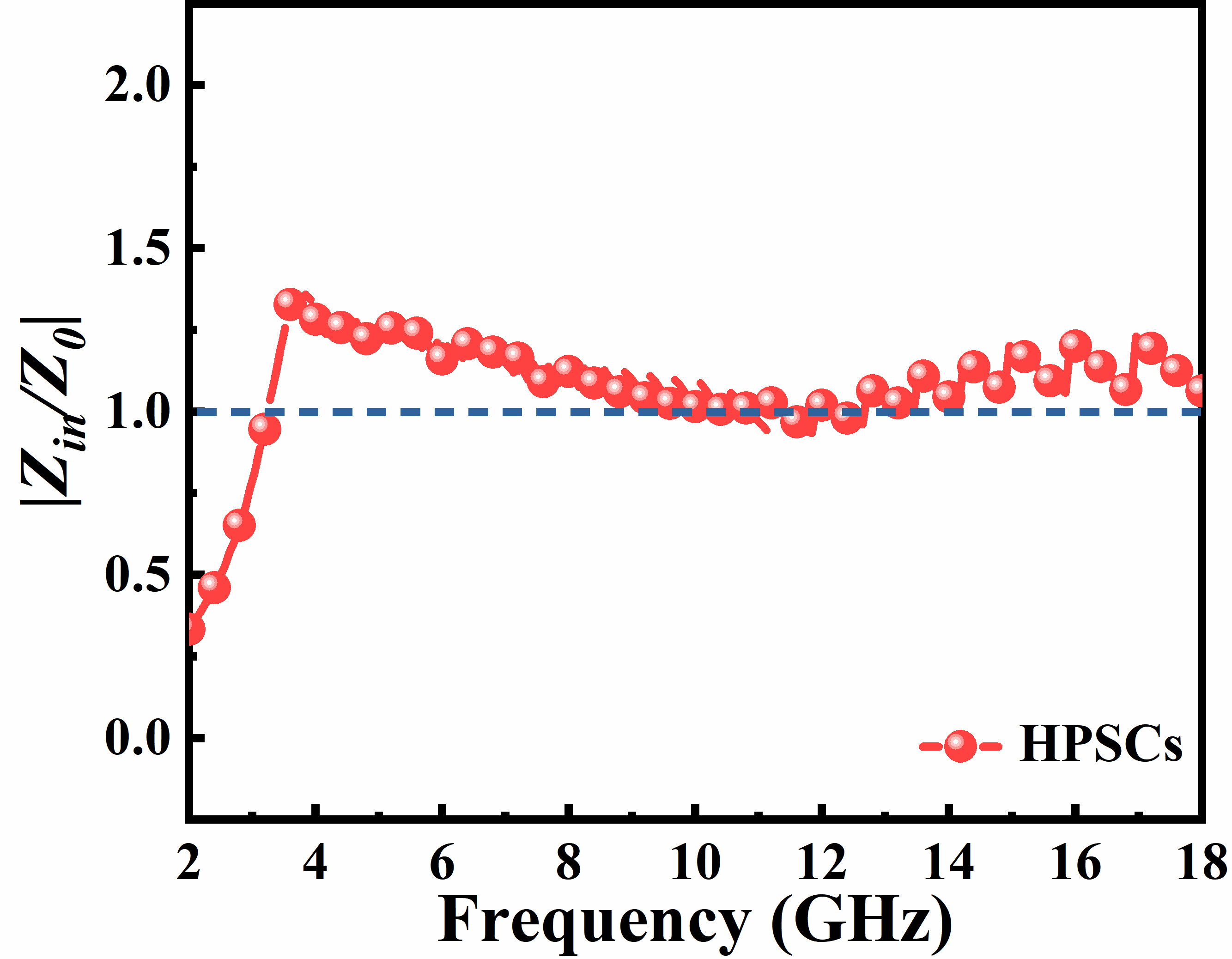


**Figure S7.** The |*Z_in_/Z_0_*| curve of HPSCs.

**Table S1.** The MA properties of similar Si-based materials

| Absorber | Mass ratio (%) | The optimal RL | | | EAB (GHz) | Ref. |
| --- | --- | --- | --- | --- | --- | --- |
|  |  | RL value (dB) | Frequency (GHz) | Thickness (mm) |  |  |
| Porou SiC/Si_3_N_4_ | 50 | -33 | 13.6 | 2.5 | 7.0 | 26 |
| C foam-Si_3_N_4_/ SiC | 50 | -43.2 | 4.7 | 5.0 | 4.0 | 27 |
| SiC nanowires/SiOC | - | -10 | 13.0 | 3.8 | - | 12 |
| 3D SiC/porous C foam | 60 | -29.7 | 16.48 | 1.75 | 2.58 | 28 |
| Porous SiO_2_/3Al_2_O_3_·2SiO_2_ | - | -31 | 9.1 | 5.0 | 4.2 | 29 |
| SiC/Porous C foam | - | -25.9 | 17.2 | 1.05 | 3.24 | 30 |
| SiC nanowires-SiOC | 100 | -20 | ~8.6 | 3.3 | 3.57 | 13 |
| SiOC | 100 | -46.0 | 10.8 | 2.64 | 3.5 | 14 |
| SiC_1.57_N_0.06_O_1.28_ | 80 | -43.3 | 7.6 | 4.5 | 3.8 | 31 |
| Carbon‐rich SiOC | - | -27.6 | ~14 | 1.5 | 3.5 | 32 |
| HPSCs | 50 | -47.9 | 12.24 | 2.3 | 4.56 | This work |

“-“ denotes that it’s unclear.
